# PReS-FINAL-2237: The diagnostic challenge of osteolytic bone lesions in autoinflammatory diseases: a case report

**DOI:** 10.1186/1546-0096-11-S2-P227

**Published:** 2013-12-05

**Authors:** R Caorsi, P Picco, A Buoncompagni, S Signa, F Minoia, S Federici, A Martini, M Gattorno

**Affiliations:** 1 2Nd Division Of Pediatrics, Istituto Gaslini, Genoa, Genova, Italy

## Introduction

Osteolytic lesions are the hallmark of a number of inherited (DIRA and Majeed syndrome) and multifactorial (CRMO and SAPHO) autoinflammatory diseases.

## Objectives

To report a clinical case in which bone lesions are part of the clinical picture of an additional inherited AID.

## Methods

We describe the clinical picture and the diagnostic tests performed in a 33 months old girl admitted in our hospital for recurrent episodes of arthritis and bone lesions.

## Results

A girl, born from uneventful pregnancy, at the age of 18 months presented arthritis of the left knee and low-grade fever with leukocytosis and increased inflammatory markers; the arthrocentesis revealed a small amount of corpuscular liquid with a negative cultural test. The girl was treated with i.v. antibiotic therapy with a partial improvement. In the following months she presented a worsening of the pain and swelling of the left knee, associated to stiffness, and started to complain of pain in the right knee and ankles; the treatment with on demand NSAIDS was poorly effective.

Due to the persistence of these symptoms, at the age of 29 months the child was admitted to another Hospital, where an X-ray of the lower limbs revealed the presence of an osteolytic lesion of about 2.3 × 0.7 cm, with indefinite margins and periosteal apposition, in the distal diaphysis of the left tibia. The bone scintigraphy showed a metabolic hyperactivity in the same area only. A biopsy of the lesion revealed a pattern consistent with a chronic osteomyelitis.

In the following months the girl complained of pain in the pelvis, legs and hands, with marked morning stiffness and lameness; the blood tests revealed a slight increase of the inflammatory markers. Bone marrow aspiration was negative for malignancies. A diagnosis of CRMO was pointed out. The girl was treated with steroids with a prompt good response but recurrence of the symptoms after discontinuation.

The girl was then admitted to our center. The blood test revealed a slight neutrophilic leukocytosis with elevation of acute phase reactants (ERS 39 mm/1 h, CRP 4,28 mg/dl); the X-ray of the left limb confirmed the presence of the osteolytic lesion with periostitis and the whole-body stir-MRI revealed a bilateral alteration of signal in the diaphysis and distal metaphysis of the tibia and in the left heel. In the following months she developed arthritis of the right knee right associated with low-grade fever. The right knee ultrasound, X-ray and MRI revealed the presence of intra-articular effusion with synovial vegetations; the arthrocentesis revealed corpuscular liquid with a high rate of polimorfonuclear cells and a negative cultural test. The family history revealed the presence of severe acne in the father and of recurrent episodes of arthritis in the grandmother. In light of that and of the symptoms complained, the PSTPIP1 gene was tested, revealing an E250Q mutation.

## Conclusion

This case enlightens a clinical overlap between different autoinflammatory diseases that has to be considered in the differential diagnosis. In fact the presence of osteolytic lesions has never been reported in PAPA syndrome.

## Disclosure of interest

None declared.

